# Interplay between Heightened Temporal Variability of Spontaneous Brain Activity and Task-Evoked Hyperactivation in the Blind

**DOI:** 10.3389/fnhum.2016.00632

**Published:** 2016-12-20

**Authors:** Rui Dai, Zirui Huang, Huihui Tu, Luoyu Wang, Sean Tanabe, Xuchu Weng, Sheng He, Dongfeng Li

**Affiliations:** ^1^School of Life Science, South China Normal UniversityGuangzhou, China; ^2^Institute of Mental Health Research, University of OttawaOttawa, ON, Canada; ^3^Center for Cognition and Brain Disorders, Hangzhou Normal UniversityHangzhou, China; ^4^Faculty of Science, University of OttawaOttawa, ON, Canada; ^5^State Key Laboratory of Brain and Cognitive Science, Institute of Biophysics, Chinese Academy of SciencesBeijing, China; ^6^Department of Psychology, University of MinnesotaMinneapolis, MN, USA

**Keywords:** temporal variability, spontaneous brain activity, blind, visual deprivation, ventral occipitotemporal cortex, fMRI

## Abstract

The brain's functional organization can be altered by visual deprivation. This is observed by comparing blind and sighted people's activation response to tactile discrimination tasks, like braille reading. Where, the blind have higher activation than the sighted upon tactile discrimination tasks, especially high activation difference is seen in ventral occipitotemporal (vOT) cortex. However, it remains unknown, whether this vOT hyperactivation is related to alteration of spontaneous activity. To address this question, we examined 16 blind subjects, 19 low-vision individuals, and 21 normally sighted controls using functional magnetic resonance imaging (fMRI). Subjects were scanned in resting-state and discrimination tactile task. In spontaneous activity, when compared to sighted subjects, we found both blind and low vision subjects had increased local signal synchronization and increased temporal variability. During tactile tasks, compared to sighted subjects, blind and low-vision subject's vOT had stronger tactile task-induced activation. Furthermore, through inter-subject partial correlation analysis, we found temporal variability is more related to tactile-task activation, than local signal synchronization's relation to tactile-induced activation. Our results further support that vision impairment induces vOT cortical reorganization. The hyperactivation in the vOT during tactile stimulus processing in the blind may be related to their greater dynamic range of spontaneous activity.

## Introduction

Visual deprivation alters the brain's functional development and organization in animals (Hubel, 1978; LeVay et al., 1980; Carlson et al., 1986) and humans (Pascual-Leone and Hamilton, 2001; Bavelier and Neville, 2002; Kupers and Ptito, 2014; Maidenbaum et al., 2014; Renier et al., 2014). Knowledge of vision loss induced brain plasticity and reorganization has been extended by noninvasive functional magnetic resonance imaging (fMRI). To date, studies in this area have adopted two independent approaches.

In the first approach, during non-visual task-fMRI, blind showed more activation than sighted people in early-tier visual area and higher-tier visual areas (Pietrini et al., 2004; Fiehler and Rösler, 2010). For instance, blind subjects exhibited greater activation in the ventral occipitotemporal (vOT) cortex, when performing tactile recognition of faces and objects (Pietrini et al., 2004).

In the second approach, using resting-state fMRI (rs-fMRI), blind subjects showed altered signal synchronization, such as inter- and intra- regional functional connectivity (Liu et al., 2007, 2011; Bedny et al., 2011; Watkins et al., 2012; Butt et al., 2013; Qin et al., 2013; Burton et al., 2014; Wang et al., 2014; Jiang et al., 2015). More specifically, the blind showed increased inter-regional functional connectivity (i.e., regional homogeneity) in especially the occipital and occipitotemporal areas, these regions include vOT.

The findings from blind subject task-fMRI and rs-fMRI lead us to ask whether there is a link between the blind's task-evoked hyperactivation and their reorganized spontaneous activity. More specifically, what alteration in spontaneous activity is related to hyperactivation in the blind? Besides rs-fMRI signal synchronization (e.g., functional connectivity), brain activities has another important property, namely, temporal variability (Kannurpatti and Biswal, 2008; Shew et al., 2009; Garrett et al., 2010, 2011; Kannurpatti et al., 2010; Vakorin et al., 2011; Huang et al., 2016). Recent temporal variability studies have provided insights in the human brain's dynamic function. For instance, higher temporal variability is thought to reflect an acceptance of greater range of stimuli. It is suggested that this broad stimuli range allows greater response, which is beneficial to neural system's adaptability and efficiency (Garrett et al., 2013b). However, temporal variability's potential importance has been largely ignored in studies of cortical plasticity in the blind. As a result, temporal variability's functional role of spontaneous activity in relation to the task-evoked hyperactivation remains unclear, as well as its context to visual deprivation.

The goal of this study was to bridge the gap between blind subject's resting-state and task-induced fMRI measures of cortical activities. We studied 16 blind subjects (BS), 19 low-vision individuals (LV), and 21 normally sighted controls (SC) using both rs-fMRI and task-fMRI (see Table 1 for a full description of BS and LV). For rs-fMRI, two local features were investigated: (1) local signal synchronization measured by regional homogeneity (ReHo; Zang et al., 2004); and (2) temporal variability (TV) measured by the standard deviation of BOLD signal (Garrett et al., 2010, 2011, 2013a; Huang et al., 2016). For task-fMRI, we examined activation as subjects performed tactile pattern recognition (Pietrini et al., 2004; Cheung et al., 2009; Kitada et al., 2009). In order to investigate the potential link between resting-state and task-evoked activity, we conducted inter-subject partial correlation analyses between resting-state ReHo, resting-state TV and task activations.

**Table 1:** **Characteristics of blind and low-vision participants**.

**Group**	**Gender**	**Age (y)**	**Onset (y)**	**Cause of blindness**	**Light perception**	**Snellen acuity**
BS	F	19	0	Congenital unknown disease	None	–
BS	F	21	0	Medication in pregnant	Faint	–
BS	M	18	0	Retinopathy of prematurity	None	–
BS	M	15	0	Congenital eyeball atrophy	None	–
BS	M	16	0	Congenital unknown disease	None	–
BS	M	18	0.3	Retinopathy of prematurity	Faint	–
BS	M	16	2	Retinopathy of prematurity	None	–
BS	M	23	3.5	Medication in pregnant	Faint	–
BS	M	21	4	Trauma	Faint	–
BS	M	18	4	Retinopathy of prematurity	Faint	–
BS	M	20	6	Retinopathy of prematurity	None	–
BS	M	21	6	Congenital optic nerve dysplasia	None	–
BS	F	16	10	Optic nerve atrophy	Faint	–
BS	M	21	12	Glaucoma, cataracts and optic nerve atrophy	None	–
BS	M	21	3L, 13R	Trauma	Faint	–
BS	M	23	14	Optic nerve atrophy	None	–
LV	F	20	0	Congenital cataracts	Yes	–
LV	M	18	0	Surgery	Yes	–
LV	F	19	0	Congenital unknown disease	Yes	–
LV	M	17	0	Retinopathy of prematurity	Yes	–
LV	M	20	0	Retinopathy of prematurity	Yes	–
LV	F	18	0	Persistent hyperplastic primary vitreous	Yes	–
LV	M	15	0	Congenital unknown disease	Yes	–
LV	F	18	0	Astigmatism	Yes	–
LV	M	18	0	Heredity	Yes	–
LV	M	21	0	Retinopathy of prematurity	Yes	20/333
LV	F	15	0	Birth hypoxia	Yes	20/200
LV	M	16	0	Congenital cataracts	Yes	20/125
LV	M	17	0	Congenital cataracts	Yes	20/100
LV	M	21	0	Unknown disease	Yes	20/400
LV	F	16	0	Unknown disease	Yes	20/400
LV	F	16	10	Unknown disease	Yes	–
LV	F	17	10	Optic nerve atrophy	Yes	–
LV	F	22	11	Cataracts, glaucoma, retinal detachment	Yes	–
LV	M	19	11	Compression of optic nerves by brain tumor	Yes	–

*BS, blind subjects; LV, low-vision individuals; L, left; R, right*.

## Methods

### Participants

We recruited visually impaired subjects from a school for the blind, including 16 BS (3 female; ages, 15–23) and 19 LV (9 female; ages, 15–22; see Table 1 for a full description of BS and LV). They were from 8th grade to 11th grade. Before they came to school, they had to pass several tests to ensure that they have normal intelligence. All of them studied braille at school. We also recruited 21 sighted controls (SC; 8 female; ages, 17–21), which are educationally matched with blind subjects. Between these groups, there was no significant difference in age (*F* = 1.47, *p* = 0.240) or gender (*F* = 1.60, *p* = 0.210) by one-way ANOVA. BS was further divided according to the age of onset of blindness (AOB) into congenital blindness (CB; *n* = 5; AOB: 0 years old), early blindness (EB; *n* = 7; AOB: 0~6 years old), and late blindness (LB; *n* = 4; AOB: >10 years old). Also, the LV was divided according to the visual acuity into two sub-groups, where 20/400 ~ 20/200 was defined as LV1 (level one; *n* = 15) and 20/200 ~ 20/100 was defined as LV2 (level two; *n* = 4). For the subjects, no history of psychiatric or neurological disorders was reported.

This study was carried out in accordance with the recommendations of the ethics committee of the Center for Cognition and Brain Disorders (CCBD) at Hangzhou Normal University with written informed consent from all subjects. All subjects gave written informed consent in accordance with the Declaration of Helsinki.

### Experimental materials and training

Plastic stamps were molded with six categories; human faces, human bodies, animals' profile, Braille (single word, corresponding to two characters), household objects and scrambles. Where, scrambled stimuli were created by randomly rearranging the other five categories images using Adobe Photoshop (San Jose, CA). Each category included 8 stamp varieties with a corresponding name. For example, the stamps for body category, there is a shape of body gesture molded into each one. And, each stamp was recalled by specific names of that gesture, like stretching. For the face category, each face was named by a Chinese surname, like Wang. For Braille category, each stimulus variety includes two characters. The participants were trained to recognize all the materials mentioned during a tactile pattern recognition training session. The participants were instructed to feel the material with their right thumb. The materials were given in a random sequence. After training, the participants were able to identify the object's name in 100% accuracy. The average training duration for each participant was 3 h. This session was conducted after rs-fMRI and before task-fMRI for each subject outside the scanner (see below).

### Experiment procedure

To begin, 8-min rs-fMRI scan was acquired for all participants, without any tasks. The resting-state scan was conducted in darkness. Before the resting-state scan, subjects were instructed to relax, stay awake and keep their eyes closed. Same instructions were applied to both the bind, low-vision and sighted groups. After the rs-fMRI, the participants were enrolled in tactile pattern recognition training, necessary for the task-fMRI during that week.

Within the week of training, a task-fMRI scan was performed. The same stimuli as during the training procedure were used in this session. Stimuli were presented in a block-design with a pseudorandom order. There were 10 blocks assigned to each category (i.e., face, body, animal, Braille, object, and scramble) yielding 60 blocks in total. Two task-fMRI runs were included. All blocks lasted 12 s and were followed by a 16 s inter-block-interval. Based on our behavioral pilot study, the presentation duration of each block (i.e., a single tactile stimulus) was set to 12 s, which ensured that all subjects had sufficient time to process each stimulus.

During fMRI scanning, two experimenters were instructed to stand quietly beside the scanner with minimal movement, avoiding potential artifactual signal changes due to alteration of the magnetic field. All stimuli were programmed using E-Prime and delivered via an audiovisual stimulus presentation system designed for a MRI environment. One of the experimenter received instructions from E-prime procedure via headphone and placed the materials in the participants' hand, according to the pre-defined timing. The other experimenter passed materials to the above experimenter according to a material list, which was made in a pseudorandom sequence.

Participants lay in the scanner with their arms comfortably resting beside their body and right hand flat with the palm facing upwards, outside the magnet bore. The subjects were instructed to avoid arm movements as much as possible. Using their right hand, they grasped and explored each material only using their right thumb and executed rubbing movements in succession until the experimenter took the material away. The task for the subjects was recognizing each tactile stimulus and recalls its name in their mind. Using their left hand, the subjects were instructed to use a response box, press left/right buttons on a response box using their left thumbs to indicate a yes/no answer as quickly as possible when they can identify the objects (including scramble objects). The response time was recorded by E-prime software. To ensure the subject's cooperation and alertness, the button response was monitored during the whole experiment. Also, to confirm that the participant could actually perform the task, two experimenters beside the participants monitored the subject's movements.

Right after the task-fMRI session, they performed the tactile task again out of the scanner room, and reported each stimulus's name aloud as well as reported what they thought in the scanner. The accuracy was recoded. On average, the recognition accuracy was high, at 99.4% (SD = 1.7%) for BS, 98.5% (SD = 3.6%) for LV, and SC for 99.4% (SD = 1.4%). No significant difference in accuracy was seen among these groups by one-way ANOVA.

### Data acquisition

A GE 3T (Discovery MR750) scanner with a standard head coil (8-channel) was used to acquire gradient-echo EPI images of the whole brain (TR, 2.0 s; TE, 30 ms; 37 slices; slice thickness = 3 mm; spacing = 0; field of view = 210 mm; flip angle = 90°; image matrix: 128 × 128). An rs-fMRI scan (240 TRs) and two task-fMRI scans (177 TRs for each) were acquired for all participants. High-resolution anatomical images (TR, 8.1 ms; TE, 3.1 ms; 176 slices; slice thickness = 1 mm; spacing = 0; field of view = 250 mm; flip angle = 8°; image matrix: 256 × 256) were acquired at the end of both rs-fMRI and task-fMRI. During scanning, subjects were instructed to relax, stay awake, and close their eyes.

### Rs-fMRI data preprocessing

Preprocessing steps were done in AFNI (http://afni.nimh.nih.gov/afni). After discarding the first four volumes, the functional images from each scan were aligned (head motion correction), slice timing corrected, transformed into Talairaich space, resampled to 3 × 3 × 3 mm^3^, spatially smoothed to 8 mm (full width at half maximum Gaussian blur), temporally standardized, and linear trends were removed. The data was then filtered with a band-pass filter preserving signals between 0.01 and 0.10 Hz (Fox and Raichle, 2007). The estimated six parameters of head motion and mean time series from the white matter (WM) and cerebrospinal fluid (CSF) were regressed out. To minimize unwanted partial voluming with gray matter, the WM and CSF masks were eroded by one voxel (Chai et al., 2012).

The issue of motion artifacts was addressed rigorously as minor group differences in motion have been shown to artificially create between-groups differences for rs-fMRI (Power et al., 2012). We addressed this issue in three steps. First, we calculated the indices of the amount of motion (shift and rotation) (Zang et al., 2007; Huang et al., 2016) for each subject, and then performed a one-way ANOVA among BS, LV, and SC. After this, no significant main effect was seen for either shift (*F* = 1.55, *p* = 0.220) or rotation (*F* = 2.86, *p* = 0.066). Second, the aforementioned time course of head motion was regressed out during preprocessing. Third, individual indices of the amount of motion (as well as age and gender) were used as covariates during all subsequent group comparisons.

### Local synchronization calculation

Regional homogeneity (ReHo) measures intra-regional (local) synchronization, which is the activity coupling between voxels within a region (Zang et al., 2004). Specifically, for each voxel, Kendall's coefficient of concordance (KCC) was calculated between the BOLD time series for the specified voxel and those of its 26 nearest neighbors (Zang et al., 2004; Zuo et al., 2013). ReHo analysis was performed for each subject by AFNI program: *3dReHo*, giving a voxel-wise ReHo map. Note that ReHo was calculated on unsmoothed data. We calculated Reho before spatially smoothing our data, essentially followed the analysis pipeline of previous studies (Zuo et al., 2013; Huang et al., 2016). After that, spatial smoothing was performed with a 8-mm fullwidth at half-maximum (FWHM) Gaussian kernel for the ReHo map, which was then transformed into Fisher's Z.

### Temporal variability calculation

For any given region, the blood oxygenation level-dependent (BOLD) signal's standard deviation (Kannurpatti and Biswal, 2008; Garrett et al., 2010, 2011; Kannurpatti et al., 2010) describes the temporal variability of BOLD-fMRI signals. The deviation across the time series for each voxel was calculated to yield a temporal variability (TV) map for each subject. Note that as the resting-state fMRI data were temporally filtered in the preprocessing step, the standard deviation of preprocessed signals is very similar to the power of the low frequency band (i.e., ALFF; Zang et al., 2007).

### Task-fMRI data analysis

Concatenate task runs were preprocessed without band-pass filter. Then, individual-subject general linear model (GLM) analyses were performed. Task-evoked activity was estimated using a BLOCK-model in AFNI. A voxel-wise contrast map was obtained for each subject based on the mean estimated regression coefficients for the five categories of normal stimuli (face, body, animal, Braille, and object) vs. scramble (control).

### Group level analyses for Rest-ReHo, Rest-TV, and β_task_

Whole brain one-way ANOVA was conducted to examine the group difference of Rest-ReHo, Rest-TV, and β_task_. *Post-hoc t*-tests including BS vs. SC, LV vs. SC, and BS vs. LV were performed for each measure. Unless otherwise stated, the reported activations of this standard analysis were thresholded at corrected *p* < 0.05. Specifically, the threshold at voxel-level was *P* < 0.005 with the cluster size >50 voxels, using the first-nearest neighbor clustering (above threshold voxels cluster if faces touch). The voxel size was 3^*^3^*^3 mm^3^. The threshold at cluster-level was *P* < 0.05 corrected for multiple-comparison using Monte Carlo simulation.

### Region of interest (ROI) analysis

As the aim of our study was to investigate the linkage between task-evoked hyperactivation and specific alteration of spontaneous activity in the blind, we thereby restricted the analysis to regions showing significant main effect (by ANOVA) among all above measures (Rest-ReHo, Rest-TV, and β_task_). Specifically, the ROIs were defined by the overlap regions of ANOVA-maps of Rest-ReHo, Rest-TV, and β_task_.

Inter-subject partial correlation analyses between Rest-ReHo, Rest-TV, and β_task_ were performed for each ROI across all the subjects (*n* = 56). Specifically, partial correlation analyses with 95% confidence interval based on 1000 bootstrap samples were calculated for each pair of measures (e.g., correlating Rest-TV and β_task_) by including the other measure as a controlling factor (e.g., Rest-ReHo). This yielded three correlations for each region, i.e., Rest-ReHo∝Rest-TV, Rest-ReHo∝β_task_, and Rest-TV∝β_task_.

## Results

### Local signal synchronization of resting-state activity (Rest-ReHo)

**Figure 1:**
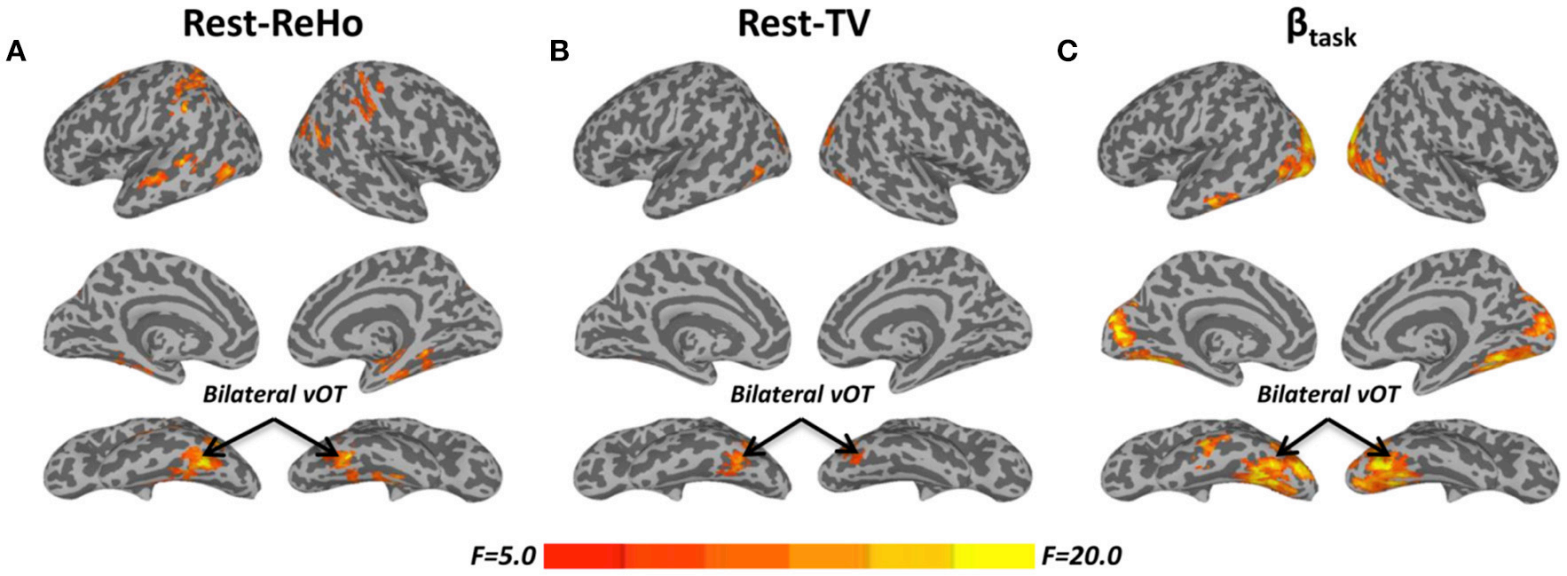
**F-maps of one-way ANOVA among blind subjects (BS), low-vision individuals (LV), and normally sighted controls (SC)**. **(A)** Significant effect of group upon resting-state regional homogeneity (Rest**-**ReHo). **(B)** Significant effect of group upon resting-state temporal variability (Rest**-**TV). **(C)** Significant effect of group upon task activation (β_task_). All F-maps were thresholded at corrected *p* < 0.05. The color bar shows voxel-wise *F*-values. The whole brain maps were surface rendered and inflated with SUMA software (http://afni.nimh.nih.gov/afni/suma) for display purposes.

**Figure 2:**
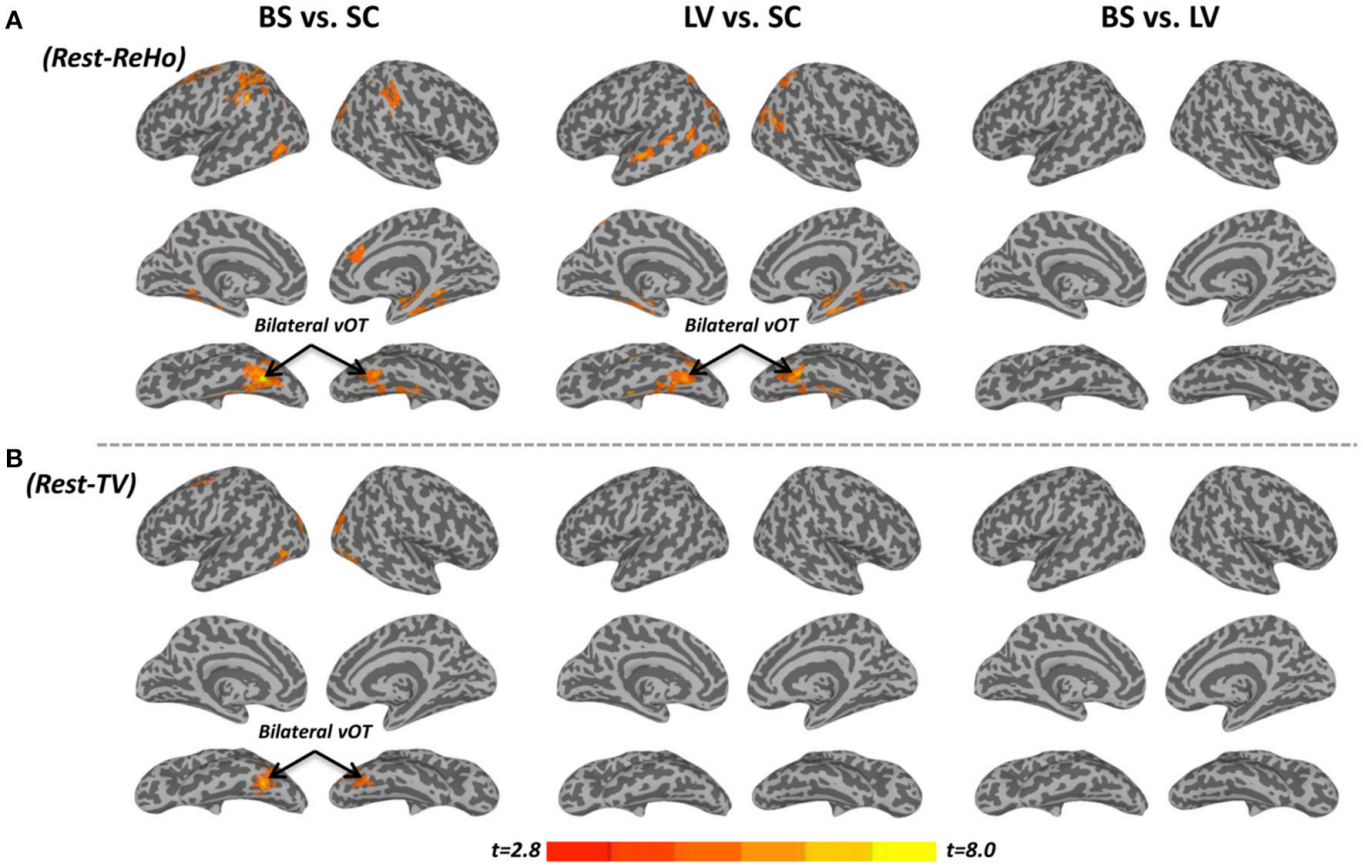
***Post-hoc t*-tests between blind subjects (BS), low-vision individuals (LV), and normally sighted controls (SC) in resting-state regional homogeneity (Rest-ReHo) and resting-state temporal variability (Rest-TV). (A)** *Post-hoc t*-tests of Rest-ReHo. **(B)** *Post-hoc t*-tests of Rest-TV. All t-maps were thresholded at corrected *p* < 0.05. The color bar shows voxel-wise *t*-values.

Intra-regional (local) synchronization can be measured by regional homogeneity (ReHo). ReHo is the coordination of activity between voxels within a region (Zang et al., 2004). In Rest-ReHo, BS, LV, and SC showed significant group effect in bilateral vOT, inferior temporal gyrus, parahippocampal gyrus, middle occipital cortex, and postcentral gyrus by a voxel-wised one-way ANOVA (Figure 1A and Table 2). These group effects were mainly accounted for by the contrast of BS vs. SC and LV vs. SC, shown by *post-hoc t*-test. Where, BS vs. SC and LV vs. SC shared similar patterns of difference (Figure 2A and Table 2). BS vs. LV showed no group difference.

**Table 2:** **Areas showing significant group differences in Rest-ReHo, Rest-TV, and β_task_**.

**Region**	**Coordinate**			**Cluster size (voxels)**
**REST-ReHo RESULTS: *F*-Test**				
Left fusiform gyrus	−25.5	−79.5	−13.5	567
Right fusiform gyrus	+41.5	−55.5	−10.5	278
Left precuneus	−7.5	−79.5	+44.5	271
Right precuneus	−16.5	+76.5	+47.5	178
Left precuneus	+25.5	+55.5	+50.5	113
Right middle temporal gyrus	−43.5	+55.5	+23.5	71
**REST-ReHo RESULTS: BS vs. SC**				
Left lingual gyrus	+19.5	+70.5	−3.5	680
Right fusiform gyrus	−25.5	+37.5	−9.5	278
Left cuneus	+25.5	+79.5	+14.5	201
Left inferior parietal lobule	+40.5	+31.5	+35.5	137
Right precuneus	−16.5	+76.5	+47.5	89
Right middle temporal gyrus	−40.5	+64.5	+14.5	58
Left inferior parietal lobule	+40.5	+31.5	+35.5	137
Right inferior temporal gyrus	−55.5	+16.5	−27.5	52
**REST-ReHo RESULTS: LV vs. SC**				
Left fusiform gyrus	+25.5	+79.5	−12.5	396
Left cuneus	+7.5	+79.5	+44.5	289
Right fusiform gyrus	−40.5	+55.5	−9.5	145
Right superior parietal lobule	−13.5	+70.5	+56.5	122
Right superior occipital gyrus	−37.5	+73.5	+29.5	88
Right parahippocampal gyrus	−37.5	+19.5	−18.5	87
Right middle temporal gyrus	−43.5	+55.5	+23.5	61
**REST-TV RESULTS: *F*-TEST**				
Left middle occipital gyrus	+31.5	+88.5	+14.5	92
Right superior occipital gyrus	−37.5	+82.5	+23.5	68
Left fusiform gyrus	+43.5	+58.5	−18.5	66
Left lingual gyrus	+4.5	+91.5	−3.5	56
Right fusiform gyrus	−46.5	+64.5	−12.5	53
**REST-TV RESULTS: BS vs. SC**				
Right superior occipital gyrus	−37.5	+82.5	+23.5	161
Left fusiform gyrus	+43.5	+58.5	−18.5	117
Left middle occipital gyrus	+31.5	+88.5	+14.5	111
Right fusiform gyrus	−46.5	+64.5	−12.5	73
**REST-TV RESULTS: LV vs. SC**				
Left middle occipital gyrus	+31.5	+88.5	+14.5	92
Right superior occipital gyrus	−37.5	+82.5	+23.5	68
Left fusiform gyrus	+43.5	+58.5	−18.5	66
Left lingual gyrus	+4.5	+91.5	−3.5	56
Right fusiform gyrus	−46.5	+64.5	−12.5	53
**β_task_ RESULTS: *F*-TEST**				
Right middle occipital gyrus	−25.5	+82.5	+14.5	2586
Left inferior frontal gyrus	+58.5	−4.5	+23.5	53
Left middle temporal gyrus	+58.5	+49.5	−9.5	50
Left precentral gyrus	+37.5	+16.5	+56.5	50
**β_task_ RESULTS: BS vs. SC**				
Right middle occipital gyrus	−25.5	+82.5	+14.5	2878
Left inferior frontal gyrus	+55.5	+19.5	−15.5	63
**β_task_ RESULTS: LV vs. SC**				
Right fusiform gyrus	−34.5	+61.5	−9.5	536
Left middle occipital gyrus	+28.5	+82.5	+5.5	406
Left inferior frontal gyrus	+58.5	−4.5	+23.5	83
Left middle temporal gyrus	+55.5	+49.5	−9.5	80
Left precentral gyrus	+37.5	+16.5	+56.5	78
Right precuneus	−13.5	+46.5	+32.5	63
**β_task_ RESULTS: BS vs. LV**				
Left cuneus	+10.5	+88.5	+20.5	673
Left fusiform gyrus	+25.5	+61.5	−6.5	236
Right fusiform gyrus	−28.5	+52.5	−6.5	99

### Temporal variability of resting-state activity (Rest-TV)

The standard deviation of blood oxygenation level-dependent (BOLD) signal (Garrett et al., 2010, 2011) describes the temporal variability (TV) of fluctuations in BOLD-fMRI signals across time within a particular region. Rest-TV showed significant group effect in the bilateral vOT and middle occipital cortex by one-way ANOVA (Figure 1B and Table 2). These differences were mostly driven by the contrast between BS vs. SC, as shown by *post-hoc t*-test (Figure 2B and Table 2). Both LV vs. SC and BS vs. LV shows no group difference.

### Task activation (β_task_) during tactile recognition

**Figure 3:**
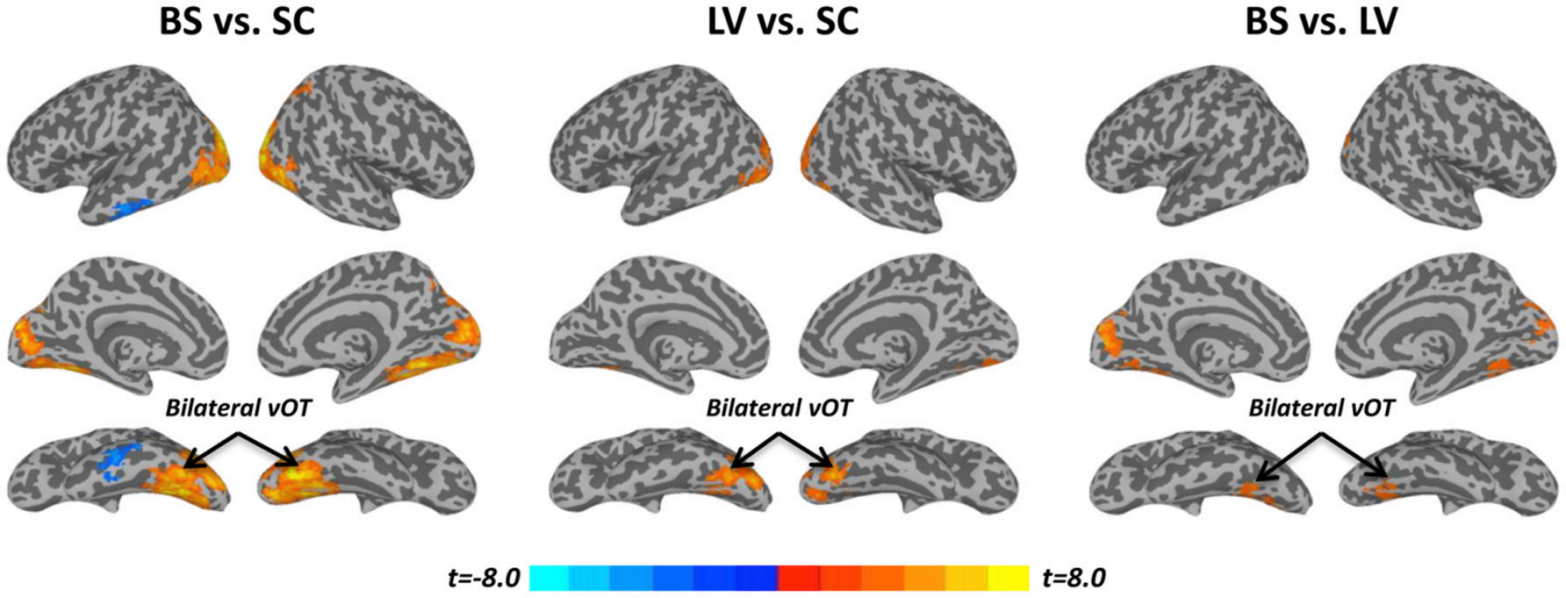
***Post-hoc t*-tests between blind subjects (BS), low-vision individuals (LV), and normally sighted controls (SC) in task activation (β_task_)**. All t-maps were thresholded at corrected *p* < 0.05. The color bar shows voxel-wise *t*-values.

Task activation (β_task_) was examined during a tactile pattern recognition task (see more details in Section Methods). Using one-way ANOVA, β_task_ showed significant group effect in wide-spread cortical regions; ventral and dorsal visual system, bilateral vOT, primary visual cortex, middle occipital cortex, and inferior temporal gyrus (Figure 1C and Table 2). With *post-hoc t*-test, evoked activity of SC to LV to BS reveals gradient increase except in the inferior temporal gyrus, where the BS showed lower activation compared to SC (Figure 3 and Table 2).

### Links between Rest-ReHo, Rest-TV, and β_task_

To examine the relationship between altered spontaneous activity and task-evoked hyperactivation in the blind, we looked at regions with significant group effects in resting-state measures (Rest-ReHo and Rest-TV) and task activation (β_task_). This was achieved by overlapping the ANOVA-maps of Rest-ReHo, Rest-TV, and β_task_. All F-maps were thresholded at corrected *p* < 0.05. The left vOT had cluster size of 125 voxels centered at [−40, −54, −9]; and the right vOT had cluster size of 28 voxels centered at [41, −54, −9]. The two vOT regions closely matched the reported processing location of fine-grained visual information in the brain (Kanwisher et al., 1997; Cohen et al., 2000; Pietrini et al., 2004; Peelen and Downing, 2005).

**Figure 4:**
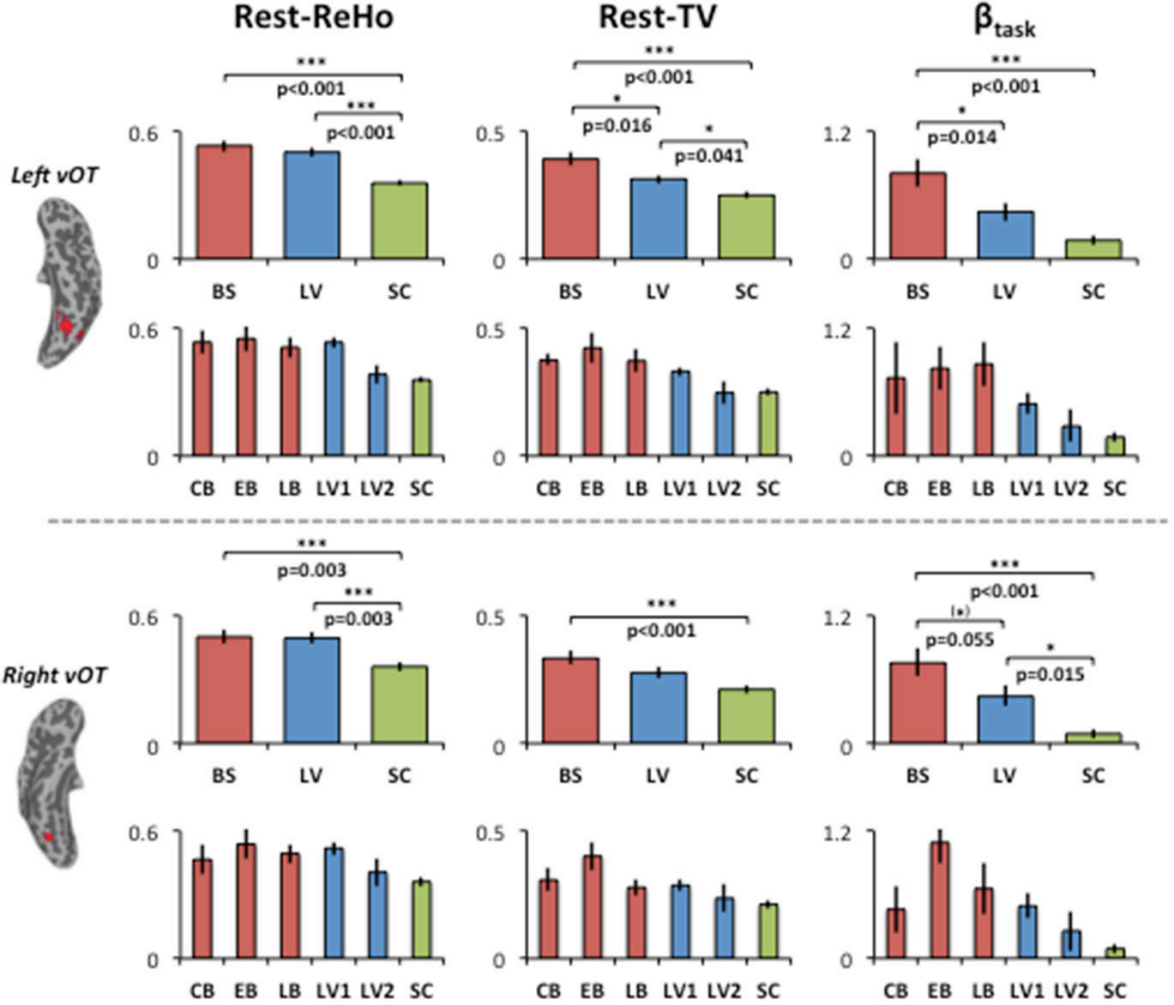
**Group contrasts of Rest-ReHo, Rest-TV, and β_task_ in the left and right vOT**. Rest-ReHo, regional homogeneity; Rest-TV, temporal variability; β_task_, task activation; BS, blind subjects; LV, low-vision subjects; SC, normally sighted controls; CB, congential blind subjects; EB, early blind subjects; LB, late blind subjects; LV1, level 1 low-vision subjects; LV2, level 2 low-vision subjects; vOT, ventral occipitotemporal cortex. ^*^p < 0.05; (^*^) marginal significance. Error bars indicate ± 1 SEM. The bar-diagrams for CB, EB, LB, LV1, LV2 and SC were used for illustration purpose. No statistical analysis was performed among these groups due to limited number of subjects.

For illustration purpose, Rest-ReHo, Rest-TV, and β_task_ group contrast in the bilateral vOT are shown in Figure 4. BS to LV to SC gradient changes were seen in all three measures, especially for Rest-TV and β_task_. To rule out the possibility that the AOB may cause BS a heterogeneous group, BS was divided into congenital blindness (CB: *n* = 5; AOB: 0 years old), early blindness (EB: *n* = 7; AOB: 0~6 years old), and late blindness (LB: *n* = 4; AOB: >10 years old). In addition, no significant correlation was observed between AOB and any of three measures (Rest-ReHo, Rest-TV, and β_task_) in the BS group (*n* = 16). Moreover, when we recruited the visual impaired subjects from the blind school, the LV subjects were all labeled as low-vision by visual acuity, which was ranging from 20/100 to light perception. To restrict the visual acuity range and to eliminate the LV group heterogeneous, we divided LV subjects into two sub-groups according to the visual acuity. Where acuity 20/400 ~ 20/200 was defined as LV1 (level one; *n* = 15) and acuity 20/200 ~ 20/100 was defined as LV2 (level two; *n* = 4). In all three measures (Rest-ReHo, Rest-TV, and β_task_), the results of LV1 group were similar to the BS group, and LV2 group were similar to the SC group (Figure 4).

**Figure 5:**
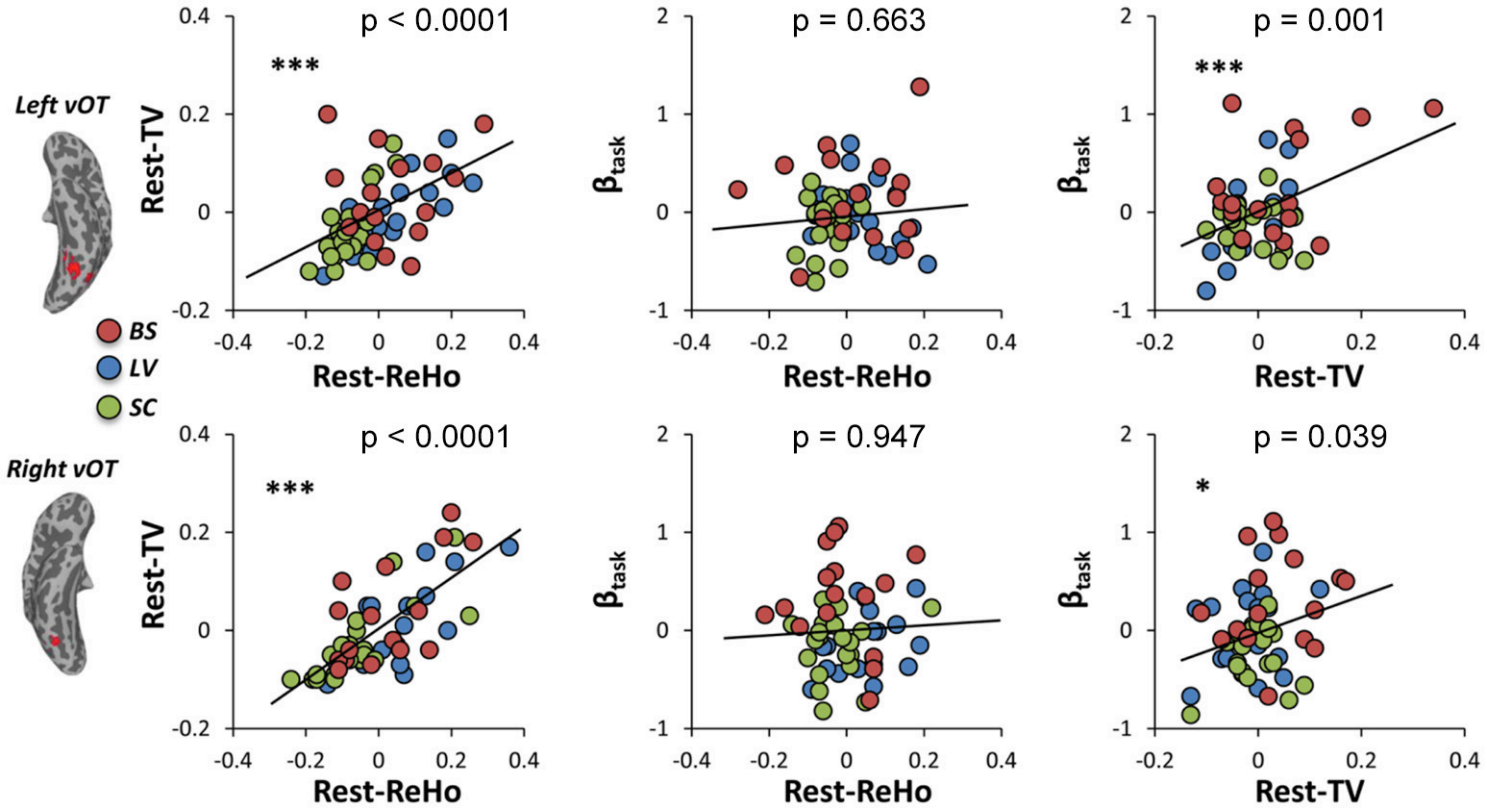
**Links across Rest-ReHo, Rest-TV, and β_task_**. Scatter plots displaying inter-subject partial correlations acorss Rest-ReHo, Rest-TV, and β_task_ in the left and right vOT (*n* = 56). For illustration purpose, the values in the x- and y- axis are residuals of a pair of variables (e.g., Rest-TV and β_task_) after regressing out the effect of the third variable (e.g., Rest-ReHo). ^***^p < 0.005; ^*^p < 0.05.

**Figure 6:**
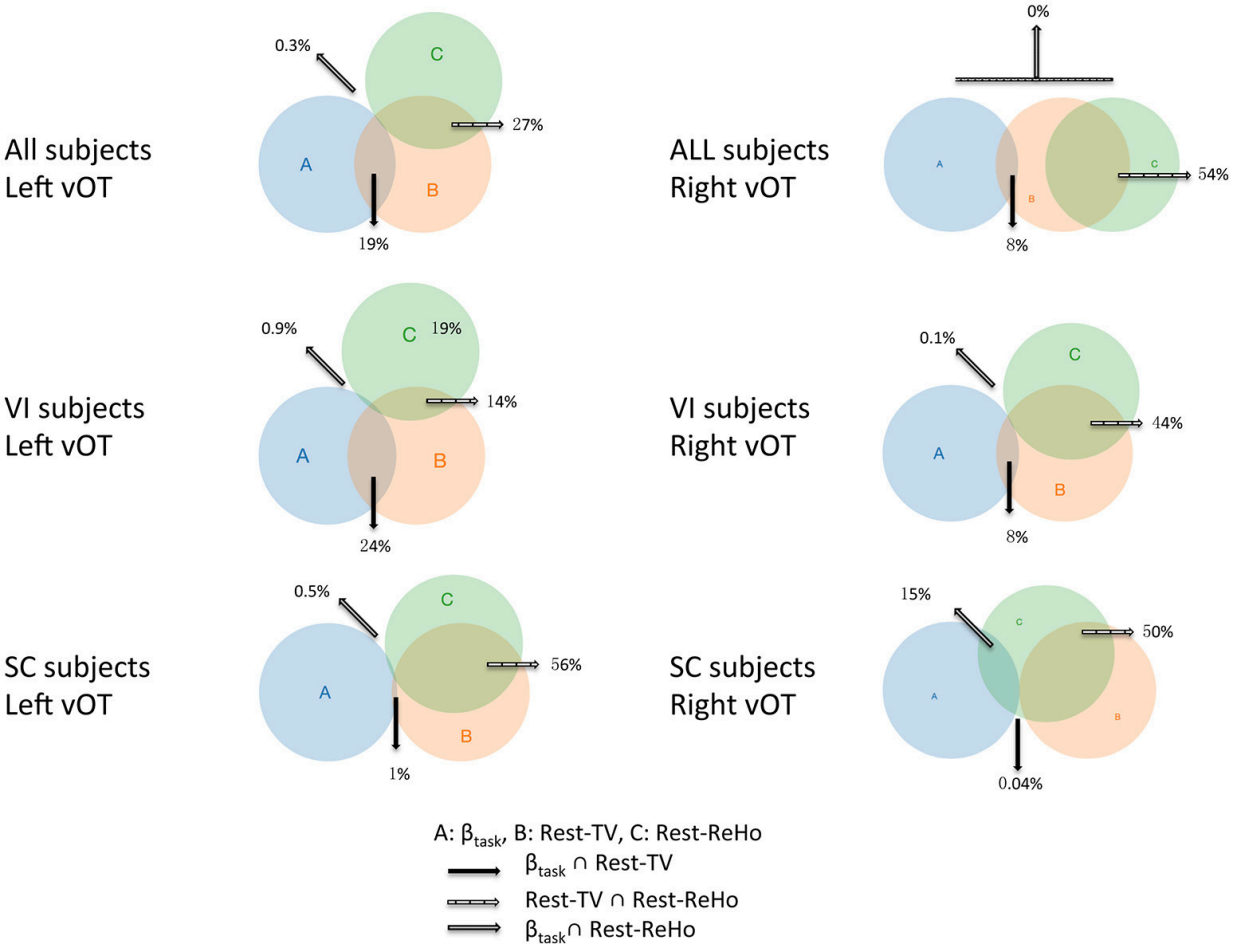
**Venn diagrams across Rest-ReHo, Rest-TV, and β_task_**. Venn diagrams displaying inter-subject partial correlations acorss Rest-ReHo, Rest-TV, and β_task_ in the left and right vOT in all subjects (*n* = 56), VI subjects (*n* = 35), and SC subjects (*n* = 21), respectively. A: β_task_, B: Rest-TV, C: Rest-ReHo.

To investigate the link between Rest-ReHo, Rest-TV, and β_task_, inter-subject partial correlation analyses (*n* = 56) were performed. Each partial correlation analysis (e.g., correlating Rest-TV and β_task_) included the other measure as a controlling factor (e.g., Rest-ReHo). This yielded three correlations for each region, i.e., Rest-ReHo∝ Rest-TV, Rest-ReHo∝β_task_, and Rest-TV∝β_task_. In both left and right vOT, significant correlations were found in Rest-ReHo∝Rest-TV (left: *r* = 0.524, *p* < 0.001; right: *r* = 0.733, *p* < 0.001) and Rest-TV∝β_task_ (left: *r* = 0.431, *p* = 0.001; right: *r* = 0.280, *p* = 0.039). Whereas, correlation of Rest-ReHo∝β_task_ was not significant in either the left (*r* = 0.060, *p* = 0.663) or right (*r* = −0.009, *p* = 0.947) vOT (Figure 5 and Table 3). The shared variances between Rest-TV and βtask were 19% (*r*^2^ = 0.19) and 8% (*r*^2^ = 0.08) in the left and right vOT, respectively (Figure 6).

**Table 3:** **Partial correlation across Rest-ReHo, Rest-TV, and β_task_**.

		**Rest-ReHo∝Rest-TV**	**Rest-ReHo ∝ β_task_**	**Rest-TV ∝ β_task_**
All subjects	L-vOT	*r* = 0.524, *p* = 0.000	*r* = 0.060, *p* = 0.663	*r* = 0.431, *p* = 0.001
	R-vOT	*r* = 0.733, *p* = 0.000	*r* = −0.009, *p* = 0.947	*r* = 0.280, *p* = 0.039
Visual impaired subjects (VI)	L-vOT	*r* = 0.369, *p* = 0.032	*r* = −0.095, *p* = 0.592	*r* = 0.489, *p* = 0.003
	R-vOT	*r* = 0.663, *p* = 0.000	*r* = −0.04, *p* = 0.821	*r* = 0.278, *p* = 0.111
Sighted controls (SC)	L-vOT	*r* = 0.745, *p* = 0.000	*r* = 0.074, *p* = 0.757	*r* = −0.102, *p* = 0.667
	R-vOT	*r* = 0.707, *p* = 0.000	*r* = −0.389, *p* = 0.090	*r* = 0.020, *p* = 0.932

Considering that the task-related activation (β_task_) in the vOT might be driven by different psychological factors (e.g., visual imagery) across BS, LV, and SC, we performed the correlation analyses separately for each of three groups (BS, LV, and SC). Unfortunately, no significant correlation between Rest-TV and β_task_ was found in either BS or LV. However, this might be due to the limited sample size of each group. Furthermore, we deem that BS and LV group were all visual impaired subjects and they were likely sharing similar neural mechanism than sighted control subjects. We thereby pooled BS and LV subjects together, namely visual impaired (VI) group (*n* = 35), and repeated partial correlation analyses again. Also, the same correlation analyses were applied to SC group (*n* = 21). We found that Rest-ReHo∝Rest-TV was significant in both VI and SC in the bilateral vOT. Rest-TV ∝ β_task_ was only significant in the VI in the left vOT, but not significant in other groups. This indicates that the correlations between Rest-ReHo∝Rest-TV and Rest-TV ∝β_task_ are fairly robust in VI. See details in Figure 6 and Table 3.

## Discussion

Using both rs-fMRI and task-fMRI, we compared 16 BS, 19 LV, and 21 SC. In specifically the vOT, we observed increased local signal synchronization and temporal variability of spontaneous activity, as well as enhanced tactile task-induced response in both BS and LV comparing to SC. Furthermore, temporal variability significantly correlates to task activation as revealed by partial correlation analyses while controlling for local signal synchronization.

Two local features of spontaneous brain activity are important for understanding functional brain organization; local signal synchronization and temporal variability (TV; Garrett et al., 2010, 2011, 2013b; Leo et al., 2012; Huang et al., 2016). Where, local signal synchronization is measured by regional homogeneity (ReHo; Zang et al., 2004). In line with a previous study (Liu et al., 2011), when comparing BS to SC, we found increased resting-state ReHo in bilateral vOT, middle occipital cortex and other brain regions. We extended these observations by showing BS had higher TV in bilateral vOT and middle occipital cortex when compared to SC. Furthermore, visual impaired subjects showed stronger evoked activity in the ventral and dorsal visual system. This is consistent with the converging evidence, the blind's early and higher-tier visual areas are activated during a variety of non-visual sensory tasks. Such as; the blind's visual dorsal stream activated during auditory spatial processing (Renier et al., 2010; Collignon et al., 2011), action control (Fiehler et al., 2009). As well as, blind's visual ventral stream activated during braille reading (Reich et al., 2011; Striem-Amit et al., 2012; Abboud et al., 2015) and object recognition (Pietrini et al., 2004; Peltier et al., 2007; Mahon et al., 2009; Amedi et al., 2010; Striem-Amit and Amedi, 2014).

Interestingly, Rest-ReHo, Rest-TV, and β_task_ group effects (ANOVA-maps) were overlapped at exactly the bilateral vOT, which closely match the reported processing foci locations for fine-grained object information. Examples of fine-grained object information are; face, visual word form, and other object categories (Kanwisher et al., 1997; Cohen et al., 2000; Peelen and Downing, 2005). Accumulating evidence indicates that vOT was not simply engaged in representing visual images, but rather, more abstract features of object form, independent of sensory modalities (Pietrini et al., 2004; Kilgour et al., 2005; James et al., 2006; Kitada et al., 2009), since these areas were activated by both visual and tactile pattern recognition tasks in sighted people (Pascual-Leone and Hamilton, 2001; Merabet et al., 2004; Pietrini et al., 2004). Collectively, the vOT seems to be a multimodal area (Merabet et al., 2004).

Our results may suggest, vOT has different weights of engagement when processing object information from different modalities. These weights can be fundamentally altered by visual impairment or deprivation accompanied by an experience-dependent cortical reorganization. For instance, in sighted subjects, the vOT is more involved in visual processing compared to tactile. In contrast, in the blind, the object information representation in the vOT should more dependent on tactile modality due to permanent visual deprivation. For low-vision subjects, who preserve the ability of processing visual information albeit limited in low frequency of visual inputs, the weights between visual and tactile information processing in the vOT should lay in between blind and sighted subjects. As revealed by our data, both the task activation of vOT and resting-state temporal variability showed a gradient increase from SC to LV and to BS. In addition, we also found that EB showed more robust task-related activations and Rest-TV in bilateral vOT than CB. Since CB lost their eyesight from birth and did not have any visual experience, these results might suggest that the vOT, especially the right vOT, benefits from an early visual “activation” to become more active at rest and also more engaged in processing fine tactile information. Converging our evidence from spontaneous activity and task activation, we provide further support that vision loss induces cortical reorganization in vOT.

We found Rest-ReHo and Rest-TV had correlations in the vOT. That is, the higher the local signal synchronization the higher the temporal variability. Our observation echoes several recent studies on the relationship between the two using a biophysically based computational model (Wong and Wang, 2006; Deco et al., 2013; Yang et al., 2014). Their modeling results revealed that temporal variability increased as a function of increasing signal synchronization. Modeling results may serve as an initial proof-of-principle of temporal variability neural bases, as the model explicitly excludes non-neural signal sources (Yang et al., 2014). This is consistent with our current findings and recent fMRI studies, where Rest-ReHo and Rest-TV were highly correlated (Yuan et al., 2013; Huang et al., 2016).

Despite correlations between signal synchronization and temporal variability, we demonstrated that visual impaired subject's heightened Rest-TV, but not the increased Rest-ReHo, is closely related to the task-evoked hyperactivation. Meaning, the relationship between Rest-ReHo and task activation can be explained by Rest-TV. This finding is consistent with a recent study by Yuan et al. (2013). The authors also observed a correlation between Rest-ReHo and task activation during finger tapping and digit-symbol substitution, whereas the variance of Rest-ReHo can be explained by other neurovascular factors such as the amplitude of low frequency fluctuations (ALFF; Zang et al., 2007). Taken together, these observed effects may be the consequence of across-subject variable—the severity of vision loss. Nevertheless, elucidating the neural mechanism on why visual deprivation leads to increased temporal variability in vOT requires further studies.

Task fMRI-BOLD signal changes are dependent on the relationship between neuronal activity and hemodynamic response. So, when interpreting the evoked activity in the blind, it is important to understand the changes in neurovascular coupling. Therefore, whether temporal variability directly influences the amplitude of task activations, or mediated by local neurovascular coupling, remains to be determined in the future (Mennes et al., 2011; Di et al., 2013). If the latter is true, the increased Rest-TV in blind's vOT may be explained by (1) increased cerebral blood flow, cerebral blood volume, and metabolic rate of oxygen (Jiang et al., 2009; Di et al., 2013); or (2) increased capillary density, enlarged diameter of arteries, or more peripheral branches, as temporal variability was also related to regions with large vessels (Di et al., 2013).

In conclusion, our findings bridge the gap between blind subject's resting-state and task-induced fMRI measures, and provide further support that vision loss induces specific vOT cortical reorganization. In the blind, the vOT hyperactivation during tactile processing may be closely related to their spontaneous activity with greater dynamic range.

## Author contributions

RD, XW, and SH conceptualized and designed the experiment. RD, HT, and LW collected the data. RD and ZH analyzed the data. RD, ZH, XW, and SH wrote the manuscript, with assistance from ST and DL. All authors discussed the results, reviewed and approved the submitted manuscript.

## Funding

This work was supported by the National Science Foundation of China (No. 31371134 to XW, No. 31172092 to DL) and National Social Science Foundation of China (No. 11AZD119 to XW).

### Conflict of interest statement

The authors declare that the research was conducted in the absence of any commercial or financial relationships that could be construed as a potential conflict of interest.
